# Tracking Socioeconomic Vulnerability Using Network Analysis: Insights from an Avian Influenza Outbreak in an Ostrich Production Network

**DOI:** 10.1371/journal.pone.0086973

**Published:** 2014-01-31

**Authors:** Christine Moore, Graeme S. Cumming, Jasper Slingsby, John Grewar

**Affiliations:** 1 Percy FitzPatrick Institute, DST/NRF Centre of Excellence, University of Cape Town, Rondebosch, Cape Town, South Africa; 2 South African Environmental Observation Network, Fynbos Node, Newlands, Cape Town, South Africa; 3 Government of the Western Cape, Department of Agriculture, Elsenburg, South Africa; National Institute for Viral Disease Control and Prevention, CDC, China, China

## Abstract

**Background:**

The focus of management in many complex systems is shifting towards facilitation, adaptation, building resilience, and reducing vulnerability. Resilience management requires the development and application of general heuristics and methods for tracking changes in both resilience and vulnerability. We explored the emergence of vulnerability in the South African domestic ostrich industry, an animal production system which typically involves 3–4 movements of each bird during its lifetime. This system has experienced several disease outbreaks, and the aim of this study was to investigate whether these movements have contributed to the vulnerability of this system to large disease outbreaks.

**Methodology/Principal Findings:**

The ostrich production system requires numerous movements of birds between different farm types associated with growth (i.e. Hatchery to juvenile rearing farm to adult rearing farm). We used 5 years of movement records between 2005 and 2011 prior to an outbreak of Highly Pathogenic Avian Influenza (H5N2). These data were analyzed using a network analysis in which the farms were represented as nodes and the movements of birds as links. We tested the hypothesis that increasing economic efficiency in the domestic ostrich industry in South Africa made the system more vulnerable to outbreak of Highly Pathogenic Avian Influenza (H5N2). Our results indicated that as time progressed, the network became increasingly vulnerable to pathogen outbreaks. The farms that became infected during the outbreak displayed network qualities, such as significantly higher connectivity and centrality, which predisposed them to be more vulnerable to disease outbreak.

**Conclusions/Significance:**

Taken in the context of previous research, our results provide strong support for the application of network analysis to track vulnerability, while also providing useful practical implications for system monitoring and management.

## Introduction

The long-term sustainability of modern society depends on the development of general principles and heuristics that allow us to work with and manage complex processes without collapsing our own life-support systems [1]. Policy and management for complex systems are increasingly being forced away from traditional optimization approaches - as they fail - and into a paradigm that focuses on facilitation, adaptation, and resilience [2]–[4]. Typical behaviors of complex systems include amplifying and regulating feedbacks between cause and effect; non-linear responses to small perturbations; and the potential for unexpected outcomes [5]. Such behaviors can be difficult to quantify and understand, particularly when their outcomes depend on irreducible uncertainties [6]. As a result, the current cutting-edge focus of research on the vulnerability of complex systems is on understanding indicators or warning signs of collapse rather than on predicting them from first principles [7], [8].

Holling and Meffe [2] have argued that as complex production systems improve their efficiency or net production, they become increasingly vulnerable to perturbations and surprises. For example, monocultures often suffer heavily from pest outbreaks; forests that are managed for wood production may become vulnerable to fire; and many of the oceanic fisheries that have been managed for ‘maximum sustained yield’ have collapsed [9]–[11]. Holling and Meffe [2] termed the tendency for managers to collapse the system, following attempts to maximize production of a single quantity, the “pathology of natural resource management”. Despite a wealth of individual cases that support the generality of the problem, however, there are relatively few empirical studies that have rigorously tracked changes in vulnerability (or its converse, resilience) as a managed production system has attempted to maximize its outputs.

Animal production systems offer a set of case studies that are data-rich, are extremely important for economies and livelihoods, and from which vulnerability can be explored in greater depth. Cumming and Norberg [12] have argued that complex systems can be described from the complementary perspectives of asymmetries, networks, and information processing. Here we develop and apply a network approach to understanding the vulnerability of animal production systems to disease.

Where traditional mean field, metapopulation and lattice based models assumed homogeneity in social relations or contact within populations, network analysis incorporates a more appropriate treatment of connection heterogeneity [13], [14]. Networks are defined as collections of potentially interacting units (termed nodes or vertices) within a system [15]. Network analysis focuses on the nature and intensity of interactions and connections (termed links or edges) between units, rather than purely on the attributes of the units themselves. By representing complex systems as collections of nodes and links, network analysis creates models that can be analyzed using standard mathematical techniques [16]. Network analysis has wide applicability in understanding systems as diverse as research collaborations, the Internet, and trophic interactions [17]–[19], and has been used to identify similar underlying mechanisms across a range of complex systems. If network analysis can be used to articulate general principles for epidemiological management [20] these should then be applicable to understanding and managing vulnerability in other complex systems.

Few datasets include contact structure data that are both relevant to pathogen transmission in large populations and sufficiently detailed to test the relevance of the network based approach [21]. Movement records of domestic livestock and poultry often provide an exception, where meticulous monitoring can produce large, detailed databases of contacts or movements that are relevant to the potential spread of disease. In recent years, network analysis has emerged as a central method for evaluating epidemics and disease transmission in many animal production systems. Notable examples include analysis of Foot and Mouth Disease in the UK [21]–[23] and Avian Influenza in the UK [24]–[27]. These investigations into specific outbreaks have led to advances in modeling and monitoring of both disease transmission and epidemic outbreaks within domestic production industries [28]–[30].

While existing studies have applied network analysis to identify the origin of disease outbreaks or monitor vulnerability in non-infected networks, studies quantifying vulnerability in systems that have experienced actual epidemics are scarce. Robinson et al. [23] investigated UK cattle trade network evolution over time to understand the emergent properties of production systems that create vulnerability, but did not link their study to a system collapse or a disease outbreak. Rautureau et al. [31] investigated vulnerability to disease in the French swine industry, concluding that while the network displayed local or regional vulnerability, the entire network was relatively disjointed. While both studies identified a tendency for networks to self organize towards vulnerability, neither linked their findings to an actual outbreak, leaving the generality of their results unclear. Here we apply a more rigorous test by examining changes in network-derived measures of vulnerability over the five years preceding a severe avian influenza outbreak in an ostrich production system in South Africa.

### Ostrich Production in South Africa as a Case Study

We examined the vulnerability of an ostrich production system using a dataset of over 18,000 transfers of domestic ostriches between farms within the Western Cape of South Africa during the period September 2005–March 2011. The dynamics of ostrich production are complex, with efforts to increase production interacting with periods of drought, fluctuations in prices of other locally produced commodities (many farmers also produce crops and some farm sheep as well as ostriches), international demand for ostrich products, and pathogen outbreaks. Meat production, constituting about a fifth of the value of each bird, and international meat exports can be used to provide an index of overall ostrich production in this region [32]. When birds are transferred between locations they are often incorporated into holdings with resident birds, allowing mixing and contact. The industry experienced H5N2 outbreaks in 2004 and 2006; and from April 2011, an exceptionally large H5N2 outbreak resulted in 42 farms testing positive for the disease, severe economic losses, and the government paying out over 6,500,000 USD in compensation. The presence of H5N2 in this system, coupled with its significant economic growth and near-collapse, make it well suited for testing the hypothesis that the emergent behavior and structure of networks during times of increased production (or, as during the financial crisis, increases in production efficiency) can be used to track system vulnerability to disease epidemics.

### Quantifying Vulnerability Using Network Metrics

**Figure 1:**
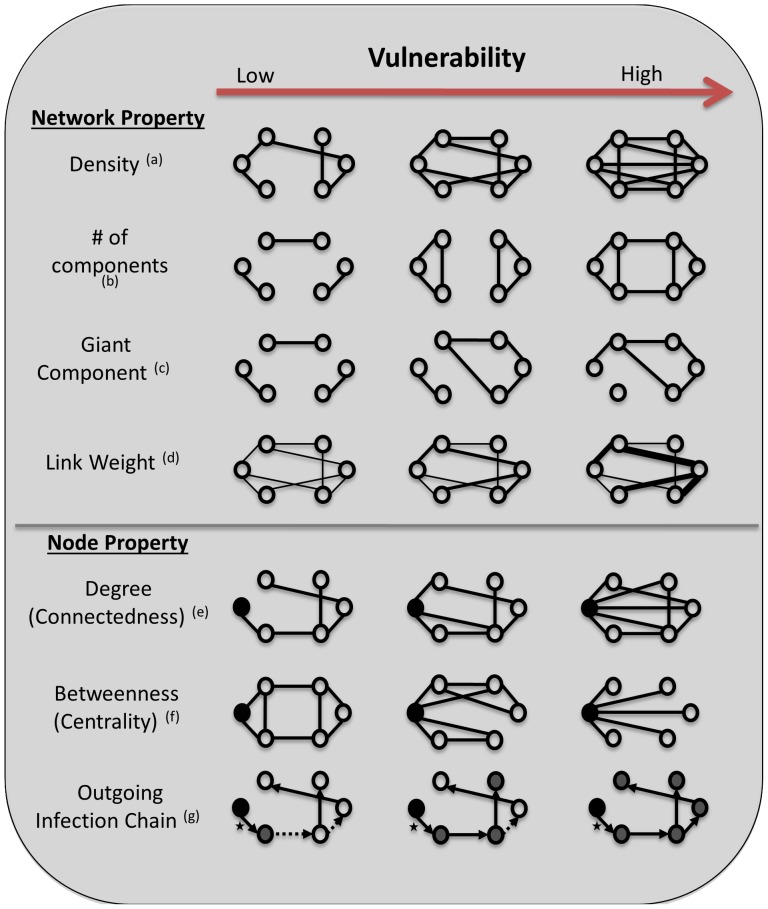
Changes in Network and Node Properties Associated with Increasing Vulnerability. *Network Properties – properties of the entire network which impact vulnerability* (a) Density (ratio of edges to nodes) – As this ratio increases, there is an increase in connections between nodes, increasing the potential paths for disease spread. (b) Number of components (number of independent/isolated sub groups) – A decrease in the number of isolated groups increases the reachability of any random node (c) Giant Component (largest independent/isolated sub group) – if the largest component contracts infection, this infection can spread further through the Giant Component compared to smaller, more isolated components which will make the whole network more vulnerable. (d) Link Weight (strength of relationship/number of units moved or frequency of movement) – As more units are moved or units are moved more frequently, there is an increase in the chance of moving infected individuals. *Node Properties – properties of each node which make the node more vulnerable or impact the vulnerability of the rest of the network. Black node = focal node* (e) Degree (number of edges) – increases in the number of edges any node has increases the vulnerability of that node to potential transmission (must have contact/connection to transfer infection) (f) Betweenness (how often a node lies on the shortest path between two other nodes) – with low vulnerability the focal node has low betweenness and for the highest vulnerability the focal node has maximum betweeness, where every node must go through the focal node to reach any other node. (g) Infection chain (the number of nodes that can be reached by the focal node, accounting for timing of movements) – the focal node has a single edge (*), and those edges in the network which are formed before this edge (i.e. those animal transfers which happen before the focal node formed) cannot contribute to the infection chain, and are depicted with a dashed line. Edges which form after this edge are depicted with a solid line. With the lowest vulnerability, the focal node can only form a chain of one, because the outgoing edge from the next node has already formed. With medium vulnerability, the subsequent edge is formed after the focal node’s edge, thus stock could move from the focal node to 3 other nodes, giving it an infection chain of 3. In the final figure all movements occur subsequent to the formation of the focal node and thus the infection chain for this node increases to 5.

The wide range of metrics used to measure properties of networks can be broadly split into network level properties (Figure 1a–d), which focus on system-wide relations and structures, and those measured at the node level (Figure 1e–g). In-depth understanding can be gained by comparing network and node measures of vulnerability over time.

The most straightforward property measured at the network level is its density, which gives a relative measure of numbers of edges to numbers of nodes (Figure 1a). Edge density captures the proportional network-wide frequency of interactions, which in turn provides information about the transmission potential of a density-dependent pathogen [33]–[35]. Increased edge density (without an increase in network size) is likely to coincide with greater vulnerability to pathogen outbreak because the number of routes for disease transmission has increased. Edge weight (Figure 1b) can also impact on network vulnerability with greater weights, representing relationship strength (ie. higher numbers of units such as number of ostriches moved) and/or the frequency of contact (ie. number of shipments of ostriches/units), creating increased vulnerability [36].

Real world networks are often composed of multiple connected subgroups, called ‘components’, that are isolated from other components. The number of components greatly affects vulnerability to diseases where transmission is based on direct contact between individuals, because nodes in components that bear no disease cannot become infected (Figure 1c). In addition, larger components can allow rapid transmission among nodes, resulting in more extensive epidemics. Some studies have found that nodes congregate preferentially in a particular component that then becomes significantly larger than the others, and have labeled it the Giant Component (Figure 1d). If large component size is coupled with a low number of components within the network (i.e., the ‘all your eggs in one basket’ syndrome), the epidemic potential of a virulent disease increases [11].

Within a directed contact network (i.e. where links represent interactions that occur from one entity to another, but not in reverse, such as movements from a breeding farm to a grower), two types of component are relevant to pathogen transmission. ‘Weak components’ are connected by directed links, but not all nodes within a group need to be mutually accessible to all other nodes. In other words, if a link exists between two nodes, regardless of direction, they will be in the same weak component [21], [37], [38]. The largest weak component in a network is known as the Giant Weak Component and has been used to estimate the upper bounds of maximal epidemic size [22], [23], [39]. The second type, ‘strong components’, are connected by directed links with each node mutually accessible to every other node either directly or indirectly [21], [38]. The largest strong component in a network is known as the Giant Strong Component, and has been used to estimate the lower bounds of maximal epidemic size [22], [23], [39]. The directionality and stage dependency of movements in some production systems reduce the predictive power of the Giant Strong Component as an overall diagnostic measure of network vulnerability [39]. In this study the Giant Strong Component over all months included only four nodes, rendering it ineffectual for understanding network vulnerability. With a sequential flow of ostriches through the production system, the likelihood for reciprocated ties between farms, or fully connected groups of farms, is low. Similar observations have been made in other production systems [39].

The most important node level measures for assessing network vulnerability are those directly related to connectivity. The simplest is the ‘Degree’, or the number of links a particular node has (Figure 1e), while ‘Betweenness’ looks at the number of times the focal node falls on the shortest path between any two other nodes (Figure 1f). Betweenness is often equated to the centrality of a node in a network, with higher values indicating greater centrality. Nodes with higher degree or betweenness would be expected to contract disease earlier in an epizootic or epidemic [9], [40].

Contact diseases follow a temporally structured chain of infection, and thus to study them adequately, both direction and the temporal sequence of interactions must be considered. One of the main criticisms of using strong and weak components to infer susceptibility of disease vulnerability is their lack of temporal information [39]. Dube et al. [39] and Noremark et al. [29] have developed methods to assess the temporal elements of ‘infection chains’ in networks (Figure 1g). Any node has the potential to have an ingoing infection chain, by which it becomes infected, and an outgoing infection chain, by which it infects other nodes. Ingoing infection chains examine the series of contacts leading into a particular node, including both the direction and relative timing of links, indicating how many nodes could have infected the focal node. Similarly, the outgoing infection chain assesses all chains leaving the focal node (considering the timing and direction of ties), indicating which other nodes the focal node could have infected [29], [38], [39].

The application of many network level measures of vulnerability is well established [17], but few studies have tested their relevance using data leading up to an actual outbreak. Comparing network measures of vulnerability to node level measures for infected versus non-infected nodes over time allows the relationships between network structure and vulnerability to be tested directly. Both network and node level measures are affected by connectivity and centrality and are expected to co-vary if they are adequate measures of vulnerability. If infected nodes are found to be more central and more connected than other nodes, this provides verification of both node and network measures of vulnerability. If these measures can be used to quantify vulnerability as a system is managed to optimize or maximize a single output, and if they have the potential to predict real-world system collapse, then they have enormous potential to contribute to identifying the thresholds and tradeoffs that are critical for the sustainable management of animal production systems in particular and natural resources in general.

## Results and Discussion

### Network Description

**Figure 2:**
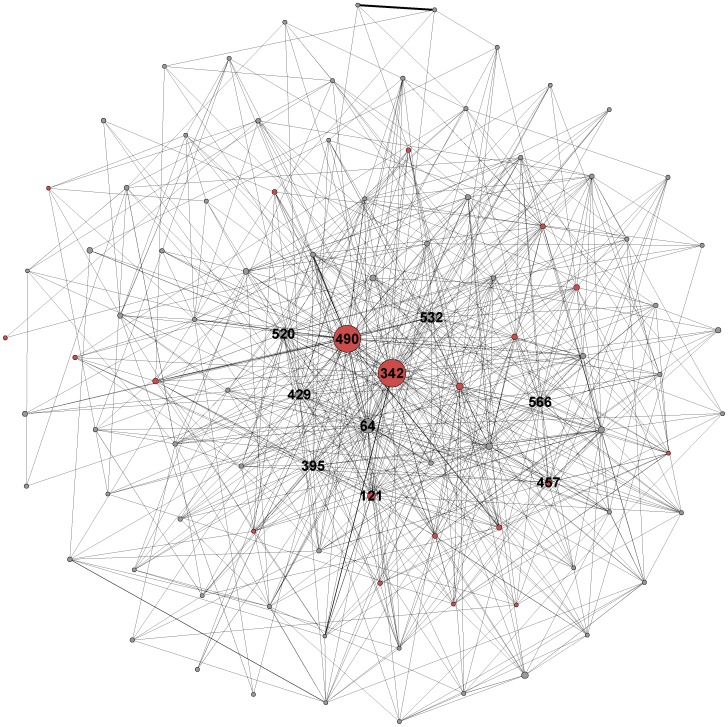
Full ostrich movement network visualization with the least connected nodes (degree <20) not shown. The farm ID numbers for the 10 most connected nodes are displayed, and all farms which tested positive for HPAI are shaded red and the farm ID number displayed for the ten farms with the highest degree.

A total of 1617 farms (nodes) participated in the Ostrich Movement Network (OMN), with 17,955 movement events (edges) involving 2,677,478 individual bird movements over the entire time series (Figure 2). Nine strong components were identified in the full network combining all data across the entire period of observation. Of these, 8 ranged from 2 to 5 nodes, while the Giant Weak Component contained 1596 nodes. While the entire network appears highly connected, greater insight can be gained by partitioning networks by time. Monthly networks displayed high variability in the number of nodes and edges, fluctuating seasonally and in a predictable fashion, as revealed by strong positive autocorrelation at 2 and 12 months and strong negative autocorrelation at 6 months when the autocorrelation function (acf) [41] is applied. The number of nodes and edges in any given month ranged from 111 to 331 interacting farms and 82 to 444 bird movements, respectively (Figure 2). Seasonal variation has also been found in other large production systems, such as swine [39] and cows [29].

### Seasonal Fluctuations and Temporal Trends in Vulnerability

**Figure 3:**
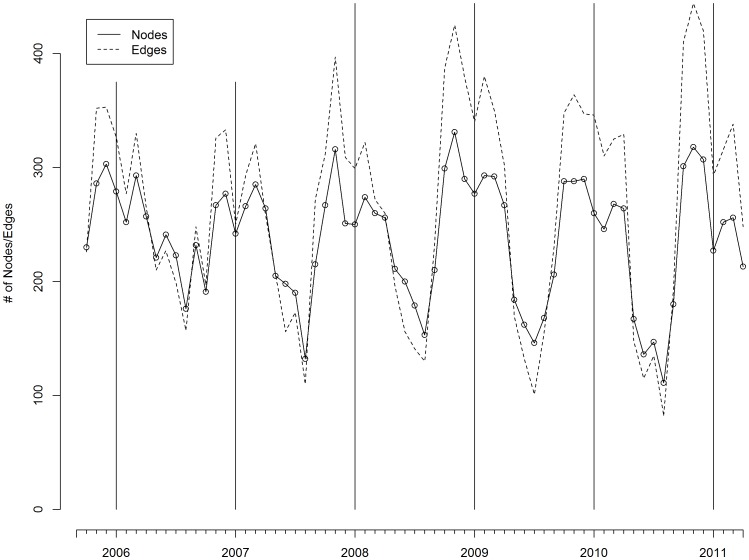
The monthly number of nodes (farms) and edges (ostrich movement events) occurring in the Western Cape, between September 2005 and March 2011. The vertical lines occur mark December of every year.

**Figure 4:**
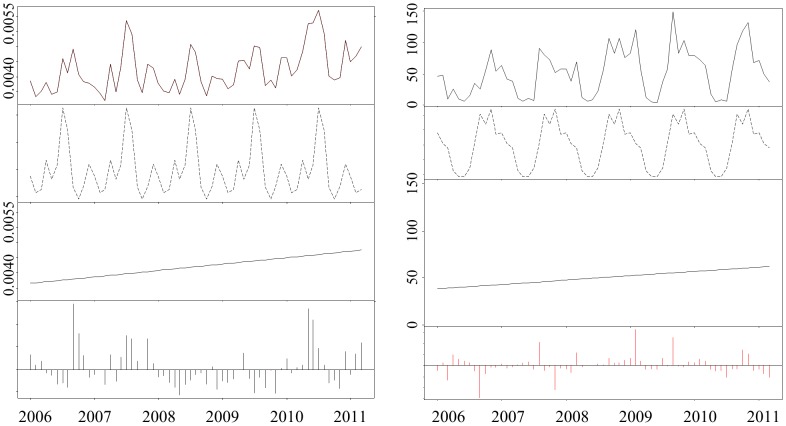
The results of a *Bfast* analysis of the (a) network density and the (b) Max outgoing infection chain. The top frame of each panel displays the network index at each time step, while the second panel depicts seasonal variation detected in the measure over time. This variation is then removed and the resulting trend is displayed in panel three. The fourth panel depicts residual variation which cannot be accounted for in the seasonal variation or trend.

**Figure 5:**
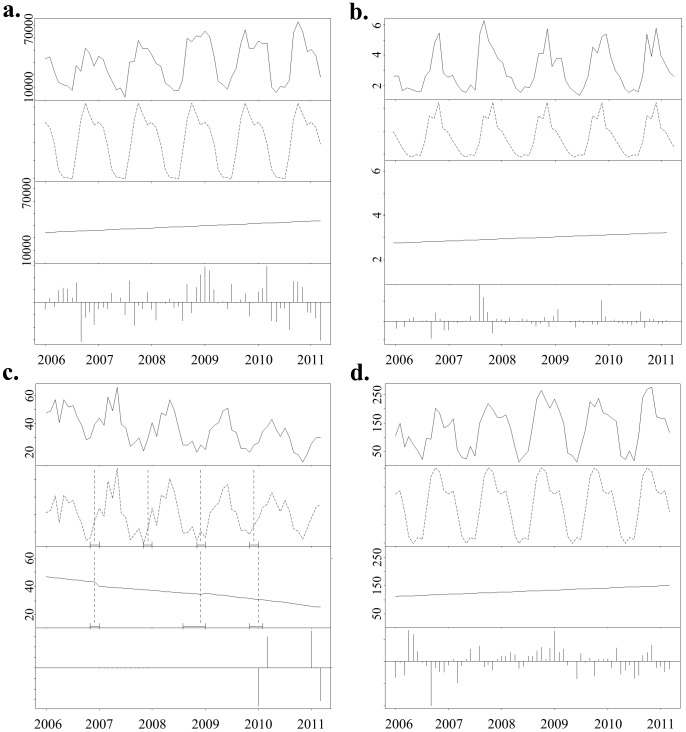
The results of a *Bfast* analysis of (a) the number of birds moved, (b) average ingoing infection chain length, (c) number of components and (d) Giant Weak Component size. The first frame of each panel displays the network index at each time step, while the second panel depicts seasonal variation detected in the measure over time. This variation is then removed and the resulting trend is displayed in panel three. The fourth panel depicts residual variation which cannot be accounted for in the seasonal variation or trend. The vertical lines in the second panel of image c indicate shifts in the season trends, while the vertical lines in the 3^rd^ panel signify an abrupt change in the trend component of the time series. The corresponding confidence interval of each shift or change is depicted by horizontal lines below each.

Analyses indicated an increase in the number of bird movements and density of connections in the movement network over the study period (Figure 3; Figure 4a). Similarly, the numbers of nodes and edges became increasingly decoupled and weak components decreased in number while the Giant Weak Component increased in size with greater numbers of ostrich movements (Figure 3, Figure 5 a & b). The reduction in compartmentalization (number of components) and the increase in connectivity (network density) reduced the system’s resilience to pathogen outbreaks and were consistent with trends identified in previous studies examining vulnerability to disease in animal production systems [21]. The increase in bird movements occurred in such a way that it aggravated transmission potential, while increasing the likelihood that an infected bird be transferred between locations before infection detected. It thus comes as no surprise that when H5N2 entered the system the initial detection of disease occurred on a single farm in April 2011, but over 40 farms became infected before the outbreak was contained four months later.

Both the maximum and average outgoing infection chains for the monthly networks increased over time, indicating that the number of other nodes that the focal node could potentially infect was increasing. The average ingoing infection chain displayed a weak decreasing trend from 2 to 1.5, although a high residual from the *Bfast* analysis leaves this finding inconclusive. The maximum ingoing infection chain did not reveal seasonal variation, displaying higher values in 2005 and 2011, but fluctuated between 5 and 15 in the intervening period. In general our findings suggest that the outgoing infection chain is more useful in quantifying vulnerability than the ingoing chain, as it is more closely coupled with seasonal trends affecting other measures and more consistent over time.

### Tracking Infection Spread

**Figure 6:**
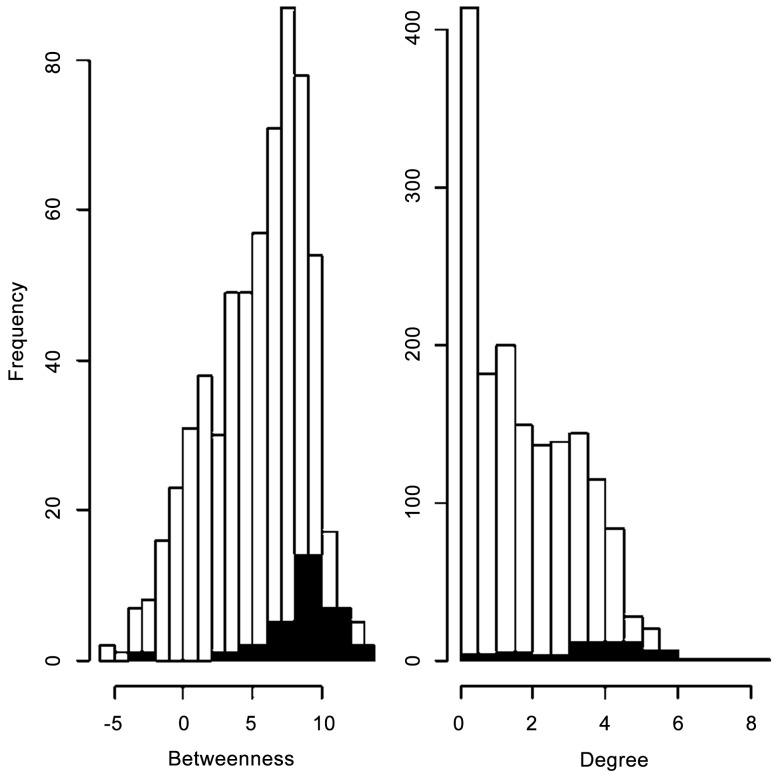
The distribution of logged betweenness (n_Infected_ = 23, n_Non_Infected_ = 324) and degree (n_Infected_ = 42, n_Non_Infected_ = 1575 scores for all farms (white) as well as the farms which tested positive for HPAI (black). A Wilcoxon signed rank test revealed that the infected farms are significantly different from infected farms in the network in both betweenness (p<0.001) and degree (p<0.001).

**Figure 7:**
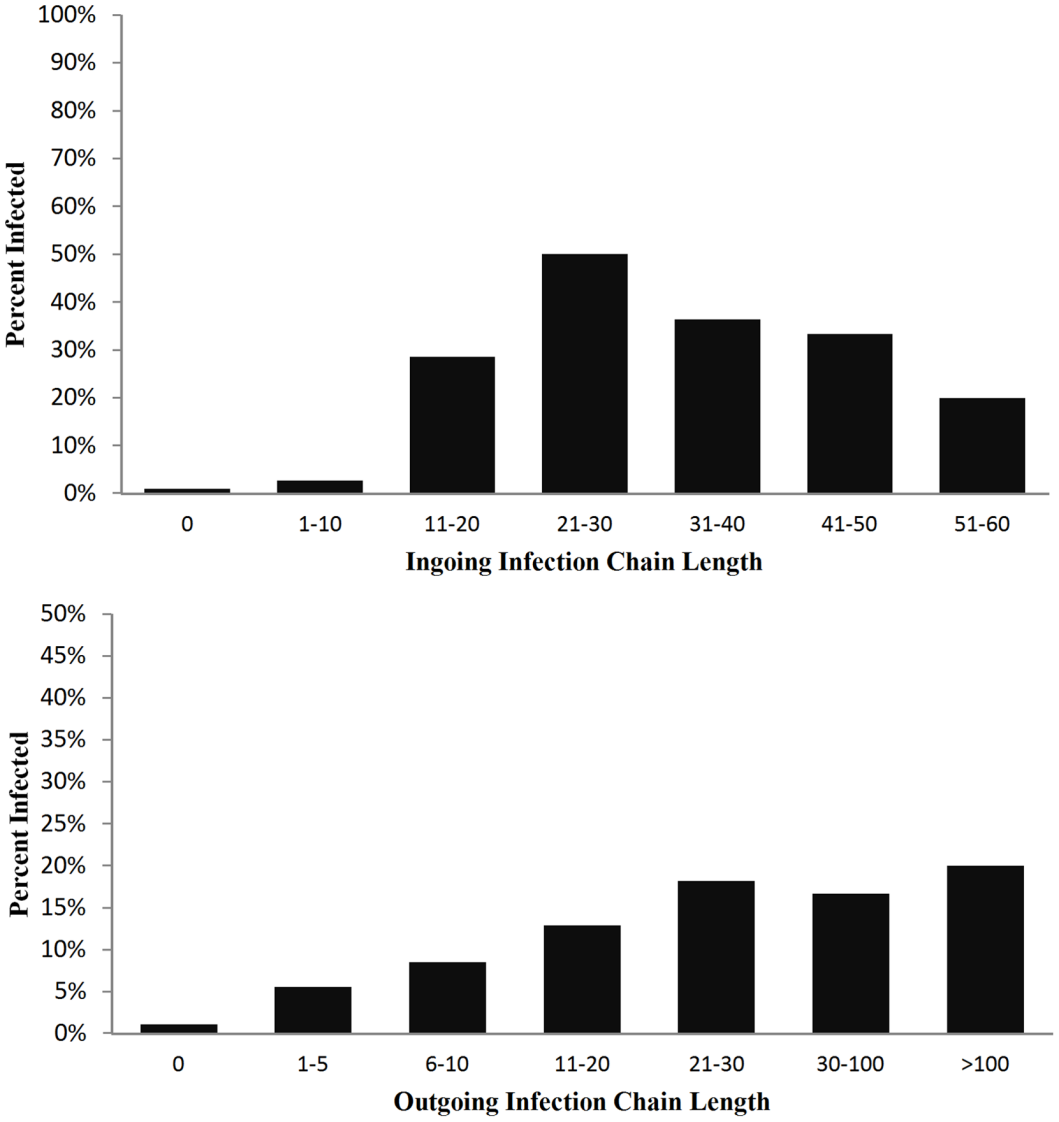
The log of the proportion of infected farms at increasing ingoing (top) and outgoing (bottom) infection chain lengths during 01/09/2010 - 01/04/2011.

Linking information on the 2011 H5N2 outbreak with changes in measures of network vulnerability through time provided a benchmark test of the utility of these measures, and allowed us to draw links between node and network level measures. Perhaps our most important finding was that the increase in network vulnerability could be quantified, via changes in network properties, using differences in node level measures between infected and uninfected farms. Wilcoxon signed rank tests for differences in betweenness and degree between infected and uninfected farms revealed that infected farms were significantly more connected (p<0.001) and more central (p<0.001) than the rest of the network (Figure 6; note that sample sizes for the tests of degree and betweenness differ due to the exclusion of a high number of nodes with null betweenness values). Outgoing and ingoing infection chain lengths for each node were calculated using a network that included all nodes and edges from September 2010 to April 1 2011, the period identified by Abolnik et al. [42] for which a form of the H5N2 virus had been in the system, and thus the movement of the virus was possible. Wilcoxon signed rank tests revealed that infected farms were more likely to have longer infection chains than those farms that did not become infected (Figure 7). Greater infection chain lengths and betweenness and degree scores for infected farms support the argument that greater connectivity increased vulnerability to disease outbreaks.

### Managing Fragility in Animal Production Systems

Our results show clearly that key attributes of the network changed in predictable ways as individual farmers attempted to maximize their profits and as the number of birds moving in the system increased. In particular, the pattern of ostrich movements made the system increasingly vulnerable to pathogen outbreaks. The potential for severe outbreaks could be reduced by implementing regulations that decrease emergent (network-level) vulnerability and/or improve monitoring to facilitate earlier detection of infected birds. Vulnerability could be reduced by constraining the direction of transfers such that farms could not exchange birds with farms from which they receive transfers. This would reduce infection chain lengths and increase the number of nodes that the disease would have to pass through to infect all farms (or Average Path Length). Another approach would be to increase the compartmentalization of the network (i.e. reducing component size) by limiting the numbers of farms that are permitted to transfer animals between one another. This would create units of interaction in which contact with members in the same neighbourhood is more likely than with farms from other neighbourhoods. While we have no data for bird interactions within farms, it is likely that increasing compartmentalization within farms (i.e. reducing contact among cohorts of birds on the same farm) would similarly reduce the spread of disease. This method would reduce emergent network vulnerability because farms that are internally compartmentalized would essentially act as multiple nodes, reducing their overall degree, betweenness and infection chain lengths.

Similarly, disease monitoring efforts are costly, logistically difficult and time consuming, limiting the number of birds and farms that can be tested. These efforts could be made more efficient if monitoring were focused on farms with high node-level vulnerability scores, and if priority were given to birds in transfers to or from farms with similarly high node-level vulnerability scores. While increased regulation is rarely popular with farmers, and might potentially incur additional costs, it would greatly reduce the vulnerability of the system to future outbreaks of H5N2 or any other pathogen introduced to the system.

### Conclusions

Our analysis supported the prediction that vulnerability to Avian Influenza outbreaks would increase as productivity was maximized in an ostrich production system in South Africa. More generally, we identified some useful principles for future analyses of network vulnerability. For instance, being able to analyze temporal trends was essential to developing a quantitative understanding of changes in vulnerability; the assembly of temporally rich data sets will be a priority for further advances in this area of research. Similarly, giant component approaches appeared less useful in quantifying vulnerability than analysis of infection chains. Infection chain analysis was developed in an epidemiological context but have parallels in many other disciplines in which network analysis is used (e.g., gene flow, pollination networks) and offer a valuable addition to this kind of analysis. Industry standards, the details of the system, and the transmission behaviors of pathogens can also have a significant impact on the usefulness of network metrics. Although some network measures, such as network density, have a wide application across most disease systems, others (such as the traits and trends of the components and infection chains) are more system-specific. Our analysis of vulnerability in an ostrich movement network, taken together with previous analyses of foot and mouth disease in the UK [21]–[23], provides a clear demonstration that network measures can be used to track vulnerability in these systems, while offering valuable insights into the design of monitoring programmes and the development of protective regulations.

## Materials and Methods

### Ostrich Production

**Figure 8:**
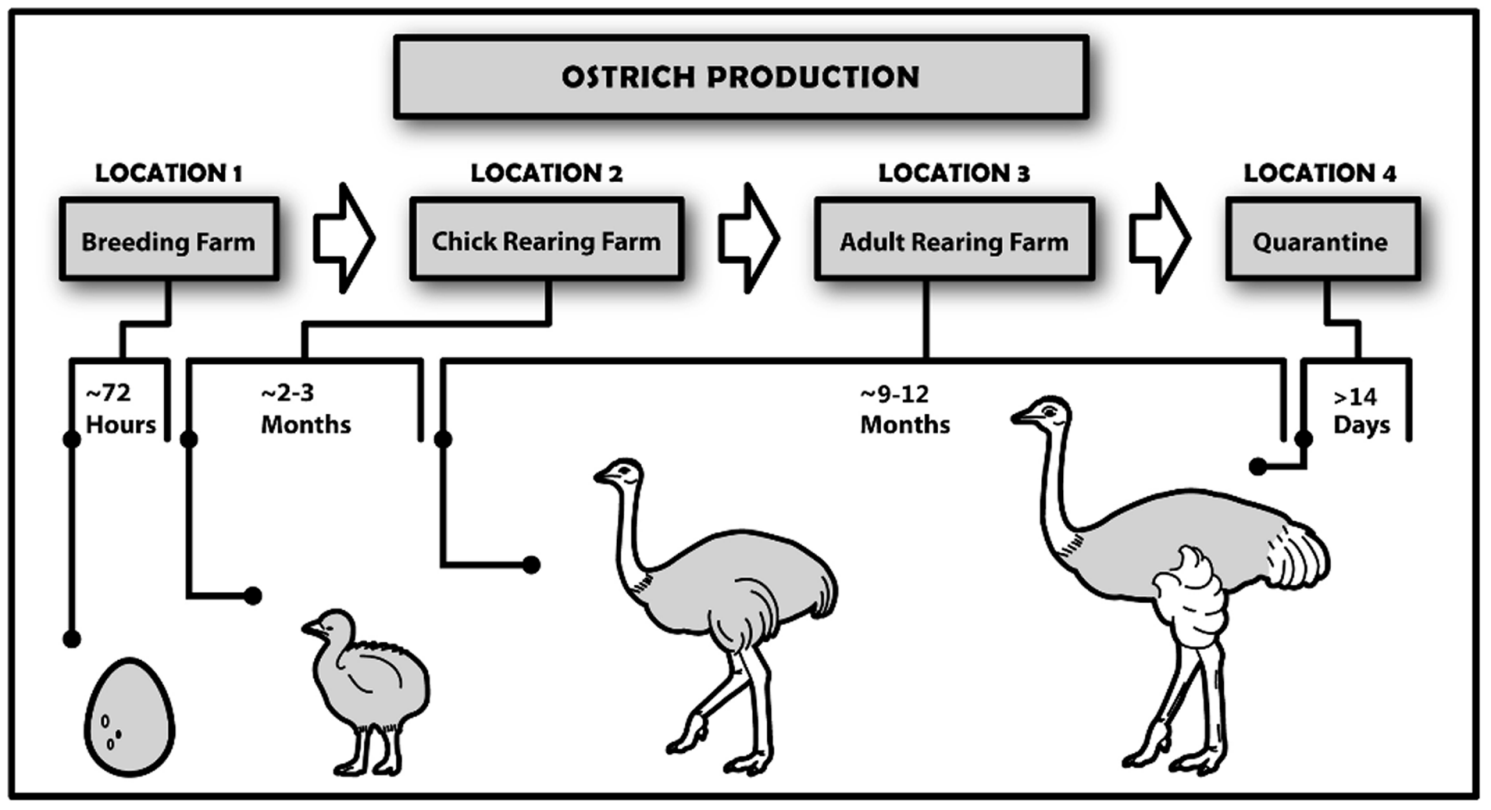
Ostrich production in South Africa. This process incorporates the movement of birds between a number of different types of farms before they are sent to the abattoir to be slaughtered. The process begins at hatcheries where eggs are incubated and, once hatched, chicks are moved to chick rearing farms within 72 hours. This most frequently occurs between September and February each year. The birds remain at these rearing farms for 2–3 months, when they are moved to adult rearing farms. They remain at these locations until they reach 70–90 kg (for approx. 12–14 months) when they are moved to quarantine farms. They remain at these farms for ∼30 days, and once deemed disease free they are transferred to an abattoir for slaughter. These last two steps of the production cycle occur primarily between September and February.

Ostrich production within the Western Cape typically involves a number of types of farm, each specializing in a specific stage of ostrich growth (Figure 8). This fragmented production system is a direct result of the excessive capital required for a farm to engage in multiple stages of production. For example, a hatchery and raiser facility could cost upwards of 100,000 USD. Additionally, it is not viable to conduct all stages of production in a single region. For example, chick raisers in more arid regions have much lower mortality rates compared with the Oudtshoorn region, where much of the feedlot-type production industry is based. As a result, it is not uncommon for a single bird to be moved upwards of 4 times during its life, residing on at least 3–4 different farms. In addition, the movement of birds is highly directional, with there being little reason for older birds to return to farms that specialize in rearing ostrich chicks or young birds.

Within each farm, ostriches are often kept in enclosures. This allows for frequent contact between new arrivals and current resident birds. Given that H5N2 is most commonly transmitted via direct contact with an infected individual, the Ostrich Movement Network (OMN) represents a plausible route of transmission. Other potential transmission pathways are via drinking water, wild birds, surfaces such as transport trucks, and the workers who accompany birds between locations [43], [44], [45]. In April 2011 the highly pathogenic avian influenza virus (HPAI) H5N2 was detected on an ostrich farm near Oudtshoorn, Western Cape [46]. By January 2012, 42 farms had tested positive for the virus. This resulted in the full eradication of stock on all H5N2-positive farms, the loss of valuable breeding stock, substantial economic losses within the region, and governmental compensation payouts in excess of 6.5 million USD.

A Department of Agriculture report [47] indicates that over the period 2001–2010 ostrich meat production ranged from approximately 6000 tonnes to just short of 10 000 tonnes per season. Although ostriches provide meat, feathers, and leather, with meat constituting only about a fifth of the value of an individual bird, meat production provides an index of overall production. An avian influenza outbreak in 2006 temporarily halted the export of ostrich meat to the European Union, where a knock on decrease in production in the 2007/2008 season was seen. A similar effect has now been experienced in the 2012/2013 season, where production decreased to less than half of the production in the 2011/2012 season (SAOBC pers comm). Available evidence suggests that ostrich movement networks continued to grow and develop during the 2008 global recession period as farmers attempted to reduce costs through such mechanisms as improving the survivorship of ostrich chicks by sending them to specialist rearing farms [43]. In attempting to either maximize production (during profitable periods) or reduce costs (during recession periods), the system thus became increasingly focused on the twin principles of efficiency and maximized yield. Efficiency is provided by the greater number of movements of birds, which results in lower costs; yield increases with the numbers of birds produced.

### Data Description

Ostrich movements in the Oudtshoorn region are recorded via permits issued by the Department of Agriculture of the Western Cape. The database of records contains the date, source, destination, batch size, as well as farmer specific information for each movement. The system has been in operation since 2005, when it was established following an outbreak of HPAI. The data available prior to September 2005 and after March 2011 were incomplete and were excluded from all analyses. The data were cleaned (i.e., screened for errors and checked against original data sheets where necessary) and a unique farm ID number was assigned to each farm in the network. The data set used only capture movements involving export farms, due to these movements being highly regulated and meticulously recorded. For the analysis it was assumed that all movements of ostriches to or from export farms are accounted for in the dataset.

### Network Construction

The movement database was used to construct an Ostrich Movement Network (OMN), which included directed ostrich movements (edge) between source and destination farms (nodes). Any two nodes were treated as being connected by a directional edge if there was at least one movement of ostriches between them during a particular month.

The movement of ostriches though out the year is not uniform, with observed seasonal variation in activity based on either the stages of the ostrich life cycle and climatic conditions. Seasonal variation is not uncommon in domestic production systems, with comparable fluctuations observed in the British livestock [22], [23] and poultry [25], [26] industries.

To observe system changes over time which related to vulnerability, movements were grouped by month, rather than day, allowing for additional network measures (i.e., density) to be examined through time. To safeguard against the potential implications of losing finer-grained temporal information that might be relevant to disease transmission and system vulnerability, these monthly sub-networks (n = 67) were analyzed using both static measures (density) and sequential measures (infection chains).

#### Time series analysis – vulnerability through time

As a proxy for production in the system, we tracked the numbers of birds being moved. To investigate system vulnerability, we used network density, number of components (strong and weak), the size of the Giant Component, average infection chain length (ingoing and outgoing) and the longest infection chain (ingoing and outgoing). All calculations were carried out using R statistical computing [48] and the packages ‘EpiContactTrace’ [29] for infection chains and *igraph* 0.5.5–3 [49] for all other network metrics. Time series were analysed using the *Bfast* analysis, implemented in the R package ‘*bfast* 2.1–1′ [50] to track network changes over the full study period despite highly seasonal fluctuations in network activity. Changes in maximum and average infection chain lengths for all nodes were tracked over time using a *Bfast* analysis. *Bfast* was originally developed for use with remotely sensed data and uses a generic change detection approach which relies on a piecewise linear model to decompose a time-series into its trend, seasonal and residual components.

#### Comparative analysis: infected vs. non-infected farms

To test whether farms which became infected during the 2011 HPAI epizootic were more connected or central than uninfected farms, their degree and betweenness were compared using Wilcoxon sign rank tests. Similar analysis was conducted for infection chain lengths (in and out) beginning at the time of infection and running for 8 months directly prior to the outbreak. We used 8 months because September 2010 was identified as the potential time of introduction of the HPAI virus in the region by Abolnik et al. [42], using molecular clock analysis based on virus DNA mutation rates.
